# Integrated Analysis Reveals That miR-193b, miR-671, and TREM-1 Correlate With a Good Response to Treatment of Human Localized Cutaneous Leishmaniasis Caused by *Leishmania braziliensis*

**DOI:** 10.3389/fimmu.2018.00640

**Published:** 2018-04-04

**Authors:** Sara Nunes, Icaro Bonyek Silva, Mariana Rosa Ampuero, Almério Libório Lopes de Noronha, Lígia Correia Lima de Souza, Thaizza Cavalcante Correia, Ricardo Khouri, Viviane Sampaio Boaventura, Aldina Barral, Pablo Ivan Pereira Ramos, Cláudia Brodskyn, Pablo Rafael Silveira Oliveira, Natalia Machado Tavares

**Affiliations:** ^1^Oswaldo Cruz Foundation, Gonçalo Moniz Institute, FIOCRUZ, Salvador, Brazil; ^2^Federal University of Bahia, Salvador, Brazil; ^3^Laboratório de Anatomia Patológica, Feira de Santana, Brazil; ^4^Centre for Data and Knowledge Integration for Health (CIDACS), FIOCRUZ, Salvador, Brazil

**Keywords:** *Leishmania braziliensis*, microRNA, skin, transcriptome, TREM-1, human leishmaniasis

## Abstract

Localized cutaneous leishmaniasis (LCL) is a chronic disease characterized by ulcerated skin lesion(s) and uncontrolled inflammation. The mechanisms underlying the pathogenesis of LCL are not completely understood, and little is known about posttranscriptional regulation during LCL. MicroRNAs (miRNAs) are non-coding small RNAs that regulate gene expression and can be implicated in the pathogenesis of LCL. We investigated the involvement of miRNAs and their targets genes in human LCL using publicly available transcriptome data sets followed by *ex vivo* validation. Initial analysis highlighted that miRNA expression is altered during LCL, as patients clustered separately from controls. Joint analysis identified eight high confidence miRNAs that had altered expression (−1.5 ≤ fold change ≥ 1.5; *p* < 0.05) between cutaneous ulcers and uninfected skin. We found that the expression of miR-193b and miR-671 are greatly associated with their target genes, CD40 and TNFR, indicating the important role of these miRNAs in the expression of genes related to the inflammatory response observed in LCL. In addition, network analysis revealed that miR-193b, miR-671, and *TREM1* correlate only in patients who show faster wound healing (up to 59 days) and not in patients who require longer cure times (more than 60 days). Given that these miRNAs are associated with control of inflammation and healing time, our findings reveal that they might influence the pathogenesis and prognosis of LCL.

## Introduction

Leishmaniasis is a group of chronic diseases caused by intracellular protozoan parasites from the *Leishmania* genus that is transmitted by infected sandflies bites (1). Localized cutaneous leishmaniasis (LCL) is the most frequent form of these diseases and is characterized by ulcerated skin lesion(s) that can take a long time to heal (2). Human LCL caused by *Leishmania braziliensis* is associated with a chronic inflammation that is critical for parasite clearance but also for tissue injury and disease pathogenesis. The notion that the severity of LCL is mainly the result of an exacerbated inflammatory response than a consequence of high parasite burden is now supported by multiple lines of evidence (1–4).

The resolution of inflammation is a tightly regulated and active process that requires a switch in gene expression, leading to a downregulation of inflammatory mediators (5, 6). Therefore, we hypothesized that regulators of gene expression could be altered during *L. braziliensis* infection, allowing a hyperactive inflammatory response even when the parasite burden is controlled. MicroRNAs (miRNAs) have emerged as key players in the regulation of gene products in recent years. These molecules are non-coding, endogenous, small RNAs (19 to 25 nucleotides) that modulate expression through the repression of translation and degradation of target messenger RNAs (mRNAs) (7, 8). miRNAs mediate posttranscriptional gene silencing or downregulation by binding to mRNAs through complete or partially complementary sequences (9). Thus, miRNAs regulate multiple biological processes through the regulation of protein translation and affect the immune response. Due to these features, miRNAs have become attractive targets for diagnosis, therapy, and biomarkers in a number of human diseases (9–12).

Altered miRNA expression has been associated with many human disorders (13–15). Regarding inflammatory skin diseases, miRNA expression profiles seem to be specific for each condition (16, 17). The upregulation of miR-203 has been demonstrated in human psoriasis lesions compared with atopic eczema skin. This miRNA inhibits the expression of SOCS-3, contributing to the dysfunction of infiltrating cells and to the pathogenesis of psoriasis (16).

The evaluation of host miRNA expression profiles in humans affected by pathogen-borne diseases can drive new strategies for diagnosis and treatment. Indeed, patients with chronic Hepatitis C Virus infection treated with Miravirsen, an antisense miRNA complementary to miR-122, showed prolonged dose-dependent reduction in viral load (18). In addition, miRNAs are promising candidates as biomarkers for sepsis (19, 20). Nevertheless, few studies have evaluated the role of miRNAs in human parasitic infections, such as *Cryptosporidium parvum* (etiological agent of cryptosporidiosis) (21–23), *Plasmodium* spp. (causative agent of malaria) (24, 25), and *Trypanosoma* ssp. (causing sleeping sickness disease) (26–29). To the best of our knowledge, this is the first report dedicated to evaluating the role of miRNAs from lesions of *L. braziliensis*-infected patients. We employed an integrative, dual-phase approach and first aimed to identify miRNAs and their target genes involved in the pathogenesis of human LCL by a joint analysis of previously published expression datasets. In a second phase, we performed *ex vivo* validation of the expression of some implicated miRNAs and correlated them with clinical features of the patients.

## Materials and Methods

### Acquisition of Microarray Datasets

Expression data sets were obtained from the NCBI^1^ Gene Expression Omnibus (GEO) database using the search terms “human,” “biopsies,” “skin,” and “*Leishmania braziliensis.”* Two microarray datasets matching these criteria were selected. The first dataset (GEO accession number GSE55664), published by Novais et al. (30), applied the Illumina HT12 v4 platform (GPL10558) to analyze 10 healthy control (HC) skin samples and 25 skin biopsies from lesions of patients with LCL from Corte de Pedra, Bahia (Brazil). The second dataset (GEO accession number GSE63931), published by Oliveira et al. (31), used the Agilent Sure Print GE Human G3v2 platform (GPL17077) and compared eight control skin samples and eight skin biopsies from patients with LCL from Itabuna, Bahia (Brazil). The Ensembl/BioMart interface^2^ was used to screen each platform for miRNA genes, which were further classified using miRBase (see Identification of High Confidence MicroRNAs). Approximately 50,000 probes were analyzed in each dataset (Figure S1 in Supplementary Material).

### Identification of High Confidence MicroRNAs

Release 21 of miRBase was used throughout our analysis. Within miRBase miRNAs are classified either as high or low confidence annotations based on standard mapped reads and deep-sequencing data (32–34). The total number of human high confidence miRNA precursors is 296, whereas 1,585 are classified as low confidence (Figure S2A in Supplementary Material). Therefore, we analyzed our data according to this miRBase standard, where miRNAs were classified as high confidence, low confidence or non-miRBase 21 miRNAs (miRNA probes were absent in miRBase release 21) in both datasets, GSE55664 and GSE63931 (Figure S2B in Supplementary Material).

### MicroRNA Expression Profiles

The expression data from both platforms were analyzed using the microarray data analysis tool Multi Experiment Viewer.^3^ Using the log_2_-transformed miRNA expression values as input, unsupervised hierarchical clustering was performed using average-linkage and Euclidean distance as metrics. Heat maps were used to represent the expression of miRNA probes through signal strength, and principal component analysis (PCA) enabled the grouping of samples based on miRNAs expression. An absolute −1.5≤ fold change of ≥1.5 with FDR adjusted *p*-value < 0.05 were used as criteria to identify differentially expressed miRNAs.

### Pathway Enrichment Analysis

Quantile-normalized data from both platforms were analyzed by Ingenuity Pathway Analysis^4^ to identify Canonical Pathways in Leishmaniasis. The “Core Analysis” was performed to relate pathways with “skin, dermis, epidermis, immune cell lines, and innate immune response” common to both Leishmaniasis datasets. After this analysis, the molecules within these pathways were searched at Target Scan^5^ as possible targets for the high confidence miRNAs differentially expressed in LCL (Figure S3 in Supplementary Material).

### Ethics Statement

This study was conducted according to the principles of the Declaration of Helsinki and under local ethical guidelines. This study was approved by the Ethical Committee of the Gonçalo Moniz Institute (Salvador, Bahia, Brazil—CAAE: 47120215.8.0000.0040). All patients provided written informed consent for the collection of samples and subsequent analysis.

### Patients and Biopsies

The patients were examined at the health post in the city of Jiquiriçá (State of Bahia, Brazil), which is a well-known area of *L. braziliensis* transmission. The criteria for diagnosis were clinical symptoms typical of LCL, histological characteristics and a positive delayed-type hypersensitivity response to *L. braziliensis* antigen or serology. Prior to therapy, biopsies were collected at the border of the lesion with a 4-mm punch from 12 patients (clinical information is available in Table S1 in Supplementary Material). Healthy skin control samples were collected from plastic surgery of seven donors living in a non-endemic area without history of LCL.

### MicroRNA Isolation

The samples were placed in TRIzol reagent (Invitrogen). Total RNA was extracted using the miRNeasy Mini Kit (Qiagen) following the manufacturer’s instructions. RNA concentrations were measured using the NanoDrop 2000 UV-Vis Spectrophotometer (Thermo Scientific). cDNA synthesis was performed using the miScript II RT Kit with HiFlex buffer (Qiagen) following the manufacturer’s instructions.

### Real-Time Quantitative PCR (RT-qPCR) Validation Assays

The relative expression of miRNAs was measured by RT-qPCR in the Jiquiriçá (Bahia, Brazil) cohort, which was composed of 12 LCL patients and 7 skin controls, as described above (see Patients and Biopsies). The assays were completed in duplicate using an ABI 7500 real-time PCR instrument (Applied Biosystems) following the manufacturer’s instructions. The expression levels of hsa-miR-155, hsa-miR-503, hsa-miR-193b, hsa-miR-99a, and hsa-miR-221 were normalized with SNORD8, SNORA70, and SNORD46 used as endogenous controls. All primers were synthesized by Integrated DNA Technologies.

### Statistical Analysis

Spearman correlation analysis tests were applied using R (for the correlation matrix) and GraphPad Prism 5 software; a *p*-value < 0.05 was considered significant.

## Results

### MicroRNA Expression Profiling Is Able to Distinguish LCL Lesions From Uninfected Skin

**Figure 1:**
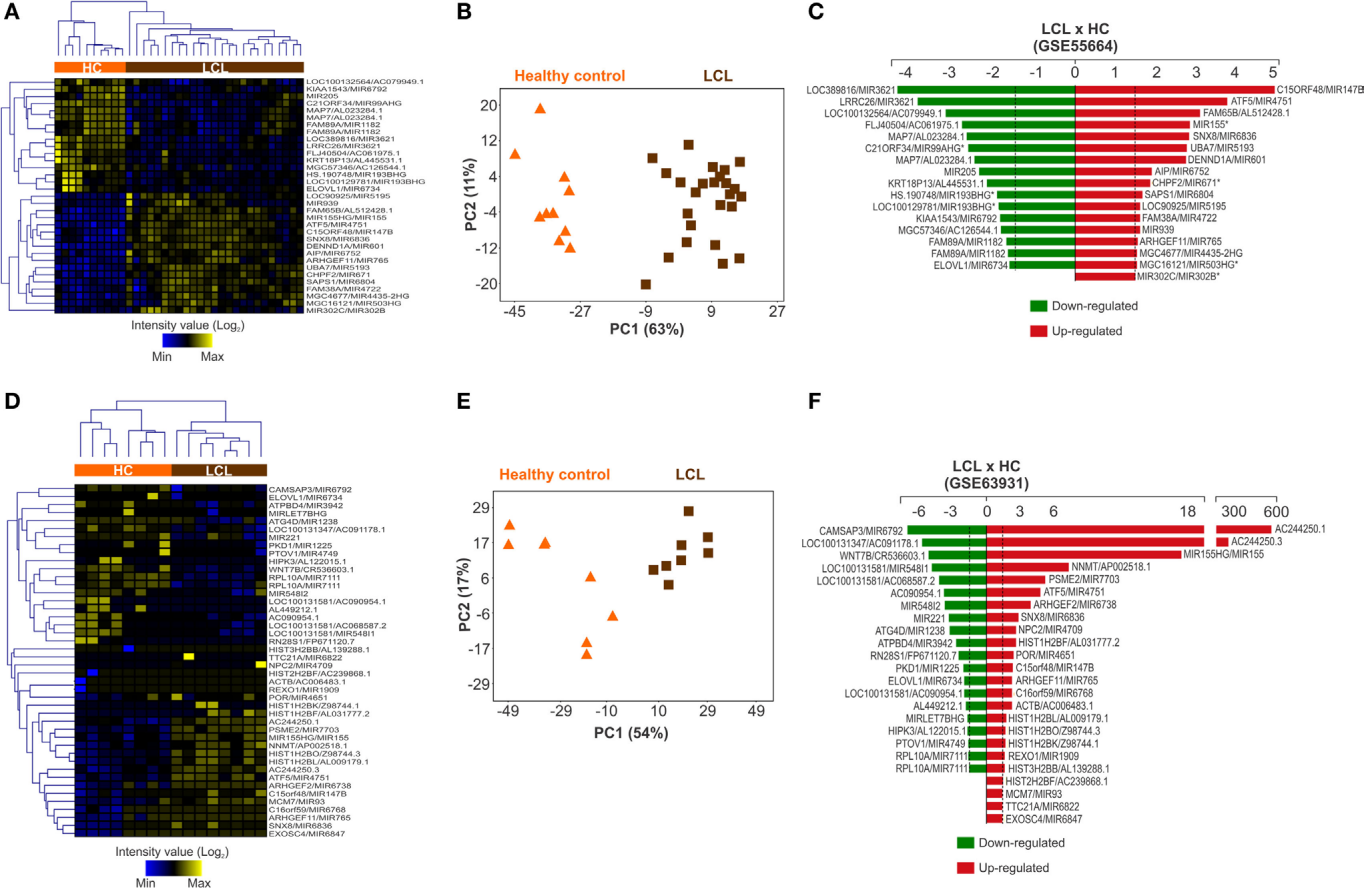
Expression profile of microRNAs is altered during cutaneous leishmaniasis caused by *L. braziliensis*. MicroRNA probes that were differentially expressed (*p* < 0.05 and −1.5< fold change >1.5) between healthy control (HC; orange bar) and localized cutaneous leishmaniasis (LCL; brown bar) skin samples were included in this analysis. Heat maps showing the expression profile of **(A)** 33 microRNA probes from GSE55664 and **(B)** 44 microRNA probes from GSE63931. Rows represent microRNA probes and columns represent samples. Unsupervised hierarchical clustering of the samples was performed using the Euclidean distance method; Principal Component Analysis using microRNA probes that were differentially expressed between the HC and LCL skin samples were conducted for **(C)** GSE55664 or **(D)** GSE63931 data sets. Each symbol represents one sample; fold changes of microRNA probes modulated in LCL relative to HC samples are shown for **(E)** GSE55664 and **(F)** GSE63931 data sets.

Two publicly available transcriptome data sets of human LCL lesions caused by *L. braziliensis* were analyzed (Figure S1 in Supplementary Material). The first (published under accession code number GSE55664) applied the Illumina HT12 v4 platform to compare global gene expression patterns between 25 skin biopsies samples from LCL patients and 10 HC samples from uninfected skin (30). From this gene array, 792 probes (from a total of 47,305) amplify miRNAs (Table S2 in Supplementary Material). The miRNA expression profile was able to separately cluster cutaneous ulcers from healthy skin biopsies (Figure S4A in Supplementary Material). For further analysis, we used 33 miRNA probes considered to be differentially regulated (*p* < 0.05; ≥1.5-fold increase or decrease in expression). The expression patterns retained the ability to segregate LCL and healthy skin samples into two distinct clusters (Figure 1A). PCA confirmed this result (Figure 1B). From these miRNA probes, 17 were upregulated (red) and 16 were downregulated (green) in LCL relative to control skin (Figure 1C).

Similar results in the same direction were obtained with the second data set (published under accession code number GSE63931) that applied the Agilent Sure Print GE Human G3v2 platform to compare eight lesion biopsies from LCL patients with eight HC skin samples (31). This gene array comprised 50,737 gene probes, with only 141 probes among these covering miRNAs regions (Table S3 in Supplementary Material). Despite this small number of miRNA probes, cluster analysis was also able to segregate ulcers from normal skin samples (Figure S4B in Supplementary Material). This clustering pattern was maintained after we restricted our analysis to the 44 miRNA probes that were differentially expressed (*p* < 0.05; fold change ≥1.5 increase or decrease in expression) between lesions and controls (Figure 1D). This pattern was also confirmed by PCA (Figure 1E). From the 44 microRNA probes, 24 miRNAs were upregulated (red) and 20 downregulated (green) in ulcers from LCL patients compared with control skin (Figure 1F). Taken together, these data show that the miRNA expression profile is broadly modulated in skin lesions caused by *L. braziliensis*.

### The Expression of 8 High Confidence MicroRNAs Are Modulated in *L. braziliensis* Lesions in Patients From Different Endemic Areas

**Figure 2:**
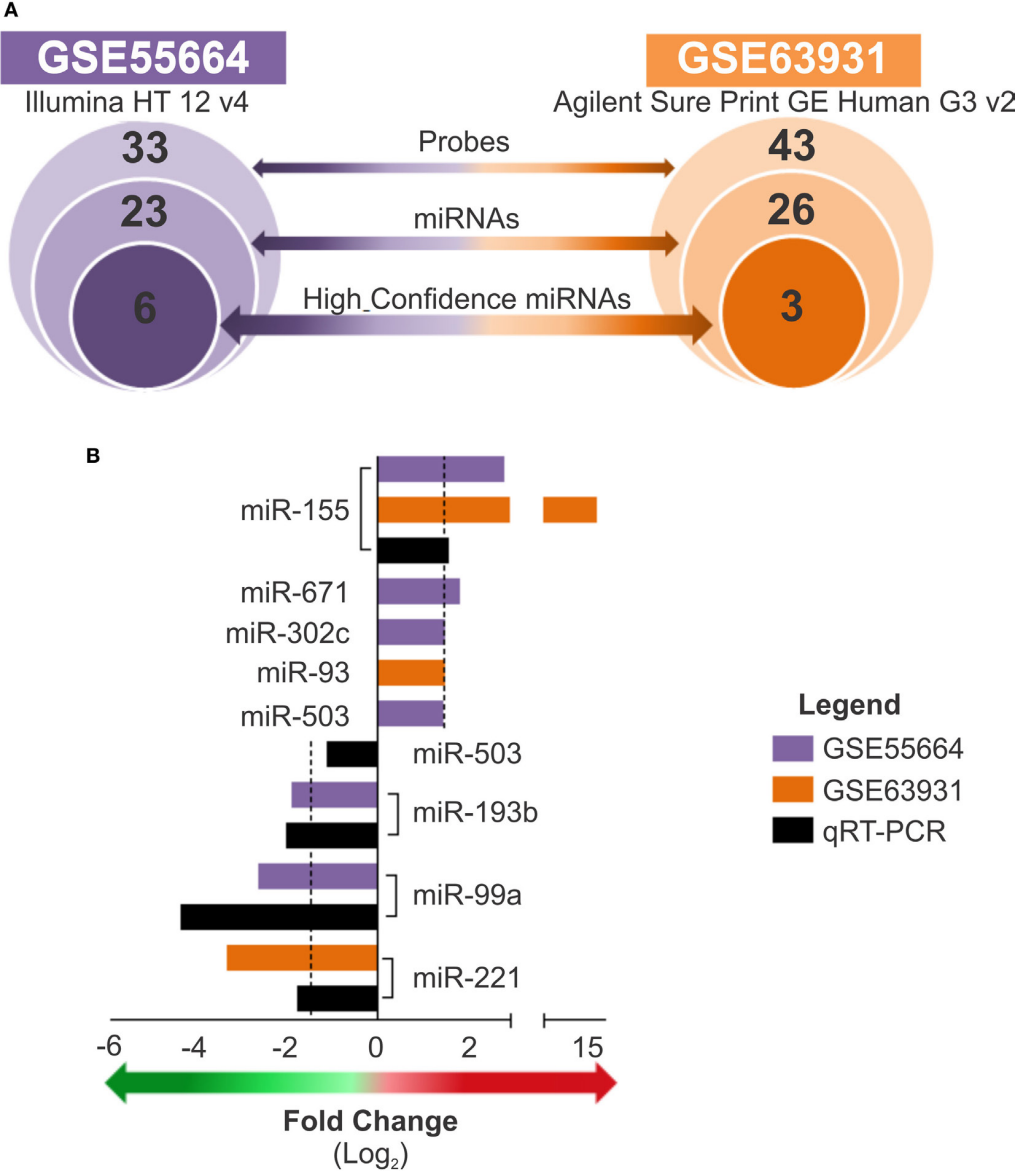
Expression of high confidence microRNAs is modulated in cutaneous lesions caused by *Leishmania braziliensis*. **(A)** The total number of probes, sequences, or high confidence microRNAs is presented for each data set, GSE55664 (purple) and GSE63931 (orange), from the outer to the inner circles, respectively (*p* < 0.05). **(B)** Bars graph shows the fold changes of high confidence microRNAs that differentially expressed from both datasets, GSE55664 (purple bars) and GSE63931 (orange bars), as well as real-time quantitative PCR to measure the relative expression of high confidence microRNAs in new samples from active lesions of patients with localized cutaneous leishmaniasis compared with healthy control skin for validation of the identified microRNAs in another cohort (black bars). The arrows indicate upregulated (red) or downregulated (green) expression (fold change ≥1.5).

A subset of high confidence miRNAs was identified based on the confidence criteria established by the miRBase database (32, 34–37). Considering the two datasets evaluated, we identified a total of 77 miRNA probes (covering 49 miRNAs) whose expression levels were modulated in LCL lesions (Table S4 in Supplementary Material); this subset included 23 miRNAs from the GSE55664 platform and 26 from the GSE63931 platform (Figure 2A). Of these, only eight were high confidence miRNAs (according to miRBase), and, among these, only miR-155 was common to both data sets. To technically validate these findings, we performed real-time RT-PCR of selected miRNAs using commercially validated assays (available for miR-155, miR-503, miR-193b, miR-99a, and miR-221) and fresh skin biopsies of cutaneous lesions from 12 patients living in another endemic area who had clinically diagnosed with LCL caused by *L. braziliensis* and seven healthy skin samples. When the fold change values between microarray data and PCR results were compared, a positive correlation (*r* = 0.7) between these platforms for the miRNAs tested was observed (Figure 2B). The differences in scales observed for each technique may be due to the sensitivity of the assays, the PCR primers used or the variable genetic backgrounds of the patients from different cohorts. Nevertheless, this result validated the altered expression of miRNAs in three different cohorts of LCL patients, and the RT-qPCR results are in accordance with our integrated analysis, which indicated that this approach is a useful tool to identify miRNA signatures.

### Identification of MicroRNA Targets in Pathways Associated With Innate Immune Response During LCL

**Figure 3:**
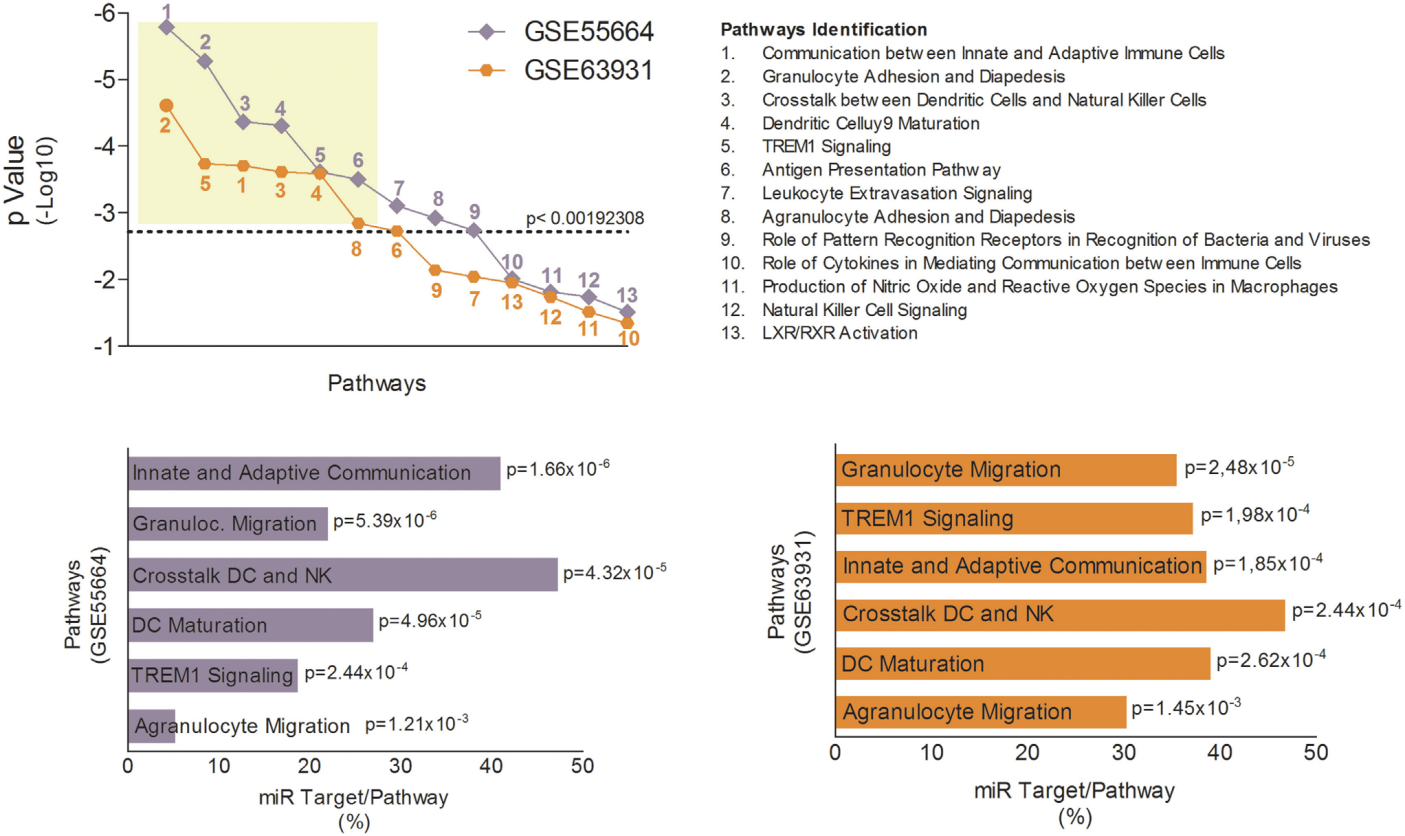
Canonical pathways associated with innate immune response identified in localized cutaneous leishmaniasis (LCL). **(A)** *p*-Value of 13 canonical pathways common to both data sets, GSE55664 (purple) and GSE63931 (orange), and related to the innate immune response. Each symbol represents one pathway identified by numbers and listed on the right. The most significant pathways in GSE66554 **(B)** and GSE63931 **(C)** are identified by *p*-value and percentage of molecules that are targets for LCL microRNAs.

The innate immune response is crucial for host defense against *Leishmania*, which needs to subvert these mechanisms to establish infection (38). Therefore, modulation of innate immune response *via* miRNAs could represent an important aspect of LCL immunopathogenesis. To identify potential genes whose mRNAs might be targeted by miRNAs during LCL, we completed pathway enrichment analysis for both datasets using Ingenuity Pathways Analysis software (Ingenuity Pathway Analysis) (Table S5 in Supplementary Material) and focusing on canonical pathways associated with innate immune response. We found 13 different inflammatory pathways (Table S6 in Supplementary Material) associated (*p* < 0.05) with LCL between both datasets (Figure 3A). However, only six of these pathways remained significantly associated with the disease after Bonferroni correction (cutoff *p* = 0.00192308). The most significantly enriched pathways in GSE55664 data set (Figure 3B) were the same as those in the GSE63931 data set (Figure 3C), reinforcing the involvement of these pathways in the pathogenesis of LCL.

Subsequently, we used the TargetScan algorithm (release 7.1) to identify genes in these pathways whose mRNAs showed consensus sequences for one or more of the eight high confidence miRNAs modulated during LCL (Figure S3 in Supplementary Material). We found, on average, that 30–40% of the molecules involved in the innate immune response against *L. braziliensis* are potential targets for the miRNAs identified here (Figure 3B,C)). Together, these finding suggest that the uncontrolled inflammatory response observed in LCL could also be due to an imbalance in the regulatory mechanisms mediated by miRNAs.

### MicroRNA-193b and -671 and Their Targets Genes Correlate With LCL Outcome

**Figure 4:**
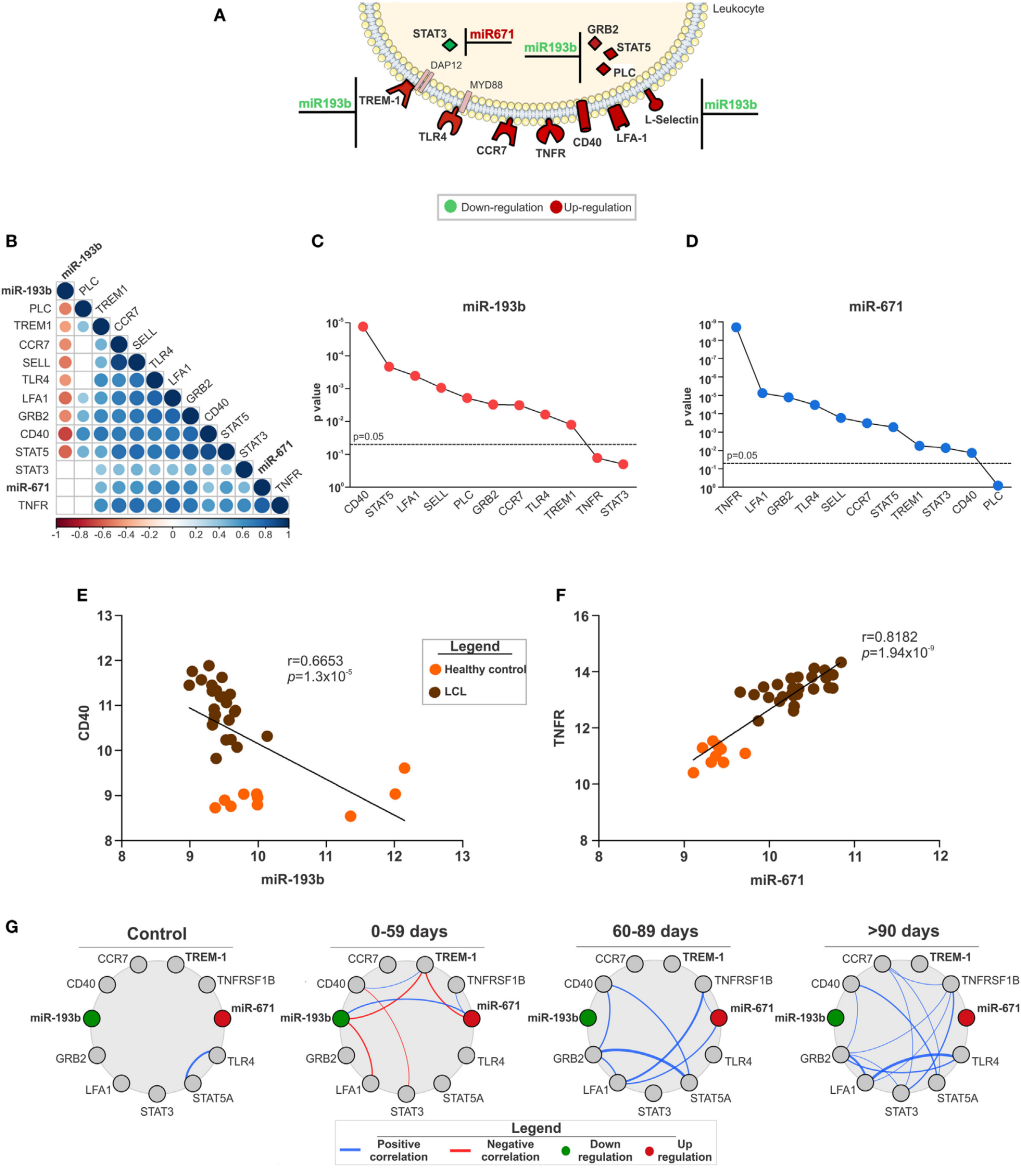
The expression of miR-193b and -671 and their target genes as key in localized cutaneous leishmaniasis (LCL) outcome. **(A)** Representative image of molecules from innate immune response pathways and their respective microRNAs with opposite expression (↑miR ↓target or ↓miR ↑target). **(B)** Correlation matrix between the expression of microRNAs and their target genes. Circles and corresponding sizes represent significance (*p* < 0.05) and *r*-values for each Spearman correlation, respectively. Colors represent the directionality of the correlation (blue infers positive correlation, whereas red indicates negative correlations). **(C)** *p*-Values of negative Spearman correlations between miR-193b and the indicated target genes are shown. **(D)** *p*-Values of positive Spearman correlations between miR-671 and the indicated target genes are described. Spearman correlations between **(E)** miR-193b/CD40 and **(F)** miR-671/TNFR. **(G)** Network analysis shows Spearman correlations (−0.6 < *r* > 0.6) between microRNAs and target genes according to the response to treatment (cured by 59, 60–89 or >90 days). Colors represent the directionality of the correlation (red line, negative correlation and blue line, positive correlation; red circles, upregulated and green circles, downregulated genes).

Specific target genes for the miRNAs were defined by expression pairing analysis performed at the probe level (Figure S3 in Supplementary Material), in addition to displaying a consensus sequence for a specific miRNA. This approach compares the expression of a miRNA and its predicted mRNA target in the same corresponding sample, which allows the identification of a potential mRNA target when its expression is inversely correlated with the expression of a given miRNA (39). Thus, only a small subset of the total genes predicted by TargetScan were defined as targets for the miRNAs (Figure 4A). In fact, six out of eight high confidence miRNAs did not have mRNA targets matching those criteria. We found only *STAT3* as a potential target for miR-671 and 11 target genes for miR-193b considering both data sets (Figure 4A).

Considering that these innate immunity pathways could be associated with tissue injury and LCL outcome, we assessed publicly available clinical information for patients in the GSE55664 data set (30). First, we correlated the expression levels of miRNAs and their target genes. The correlation matrix presented in Figure 4B) summarizes these results. Almost every gene evaluated correlated with its respective miRNA. Interestingly, only miR-193b had a negative correlation with its target genes (Figure 4B), suggesting that miR-193b could play a significant role in the amplification of the inflammatory response observed in LCL. The statistical significance (*p*-value) of the correlations between miR-193b (Figure 4C) and miR-671 (Figure 4D) with their target genes reinforced the relevance of miRNAs in the regulation of inflammation. The most significant genes (*CD40* and *TNFR*) were selected for detailed correlation analysis with miR-193b (Figure 4E) and miR-671 (Figure 4F). As expected, there was a negative correlation between miR-193b and *CD40*, meaning that increased levels of *CD40* are frequently related with reduced miR-193b (Figure 4E), which contributes to the inflammatory response. On the other hand, miR-671 showed a strong positive correlation with *TNFR* (Figure 4F), suggesting that miR-671 could indirectly enhance the expression of TNFR.

We also examined the correlations between miRNAs and their target genes within groups of patients who had different responses to treatment including patients who were cured in less than 59 days, between 60 and 89 days and more than 90 days. The correlation profile was distinct for each group of patients (Figure 4G), especially for those with reduced cure times (0–59 days). Only patients with a good response to treatment (0–59 days) presented negative correlations (red lines) among miR-193b, TREM-1, miR-671, and LFA-1. On the other hand, patients who needed more than 60 days to be cured presented only positive correlations (blue lines) between different molecules, such as GRB2 and STAT5. Moreover, the total number of correlations was distinct among these groups, increasing in groups of patients with the longest healing times (Figure S5 in Supplementary Material). In addition, there was no correlation among miR-193b, miR-671, and TREM-1 in patients who required more than 90 days of treatment (Figure 4G), suggesting a role for miR-193b, miR-671, and *TREM1* in better prognosis for LCL.

## Discussion

Integrative analysis of data sets from different sources improves the understanding of complex interactions and networks involved in disease pathogenesis (40–42). This approach allowed us to identify the expression profile of miRNAs and their target genes in lesion samples of patients with LCL caused by *L. braziliensis* from different endemic areas.

Differential miRNA expression has been investigated in different contexts of human diseases, such as biomarkers of allergic asthma (43), treatment for Hepatitis C Virus infection (18), prognostic markers of different types of cancer (44–46) and many others. However, little is known about miRNA expression profiles in human parasitic diseases. Some studies have identified profiles of circulating miRNAs in murine models of infection by *Schistosoma japonicum* (47), *Schistosoma mansoni* (48), and *Toxoplasma gondii* (49) as potential targets for disease progression or diagnosis. These miRNA expression profiles in the plasma are specific for each disease evaluated, but these findings need to be validated in human samples. On the other hand, miR-451 is significantly enriched in human red blood cells infected by *Plasmodium falciparum* (50), and, more importantly, miR-451 translocates to the parasite and protects the host, thus reducing parasite survival (51). Those findings provide the first evidence of the protective role from a specific miRNA in human malaria. Recently, it was shown that circulating levels of miR-451 and miR-16 were downregulated in patients infected with *Plasmodium vivax* at parasitic stages or with multi-organ failure involvement (25, 52). This association of miRNAs profile with clinical severity of malaria infection emphasize that miRNAs are potential tools for diagnosis and therapeutics for parasitic diseases. Moreover, 25 miRNAs released by *S. mansoni* exosomes are present at high levels in the circulation of infected mice. These results provide evidence of schistosome-derived exosomes could play important roles in host-parasite interactions and could be a useful tool in the development of vaccines and therapeutics (53).

Few studies have evaluated the modulation of miRNA expression during *Leishmania* infection in cultured cells or experimental models of infection by species causative of Visceral Leishmaniasis (54–58), species from the Old World that causes LCL (59–61) or both (62). Their findings indicate that the expression of miRNAs is specific for each condition. Only a recent study evaluated the expression of 84 miRNAs in mouse macrophages infected by*L. amazonensis* at different time points after infection (63). These authors showed that miR-294 and miR-721 are induced by *L. amazonensis* infection and target NOS2 and l-arginine metabolism, thus favoring parasite establishment. However, altered expression of these miRNAs was not observed in any of the other studies of *Leishmania* infection. Together, these studies underscore the specificity of each model, since the results obtained were not observed in any different condition tested. In fact, this reinforces the importance of evaluating alterations directly from naturally infected patients where the complexity of the human organism is taken into account and potential artifacts from experimental models are avoided.

The approach we applied in this study also allowed the identification of enriched canonical pathways among the genes modulated in each data set. Since miRNAs are important regulators of innate and adaptive immune responses in different pathological conditions (10, 64), such as autoimmune diseases (65), diabetes (66), toxoplasmosis (67), and infections (68, 69), we correlated the molecules within each pathway with the corresponding miRNA by expression pairing.

The analysis of expression pairing results in a more focused set of miRNAs since the prediction of miRNA targets identifies a great number of potential mRNAs. This approach identifies an inverse correlation between a given miRNA and its mRNA targets, which significantly reduces the number of potential mRNA candidates and has been applied in cancer studies (70). From this analysis, only miR-193b and miR-671 had significant mRNA targets that matched these criteria. The involvement of miR-193b in the regulation of relevant target genes has already been described for different types of cancers (71–73) and for hepatitis B infection in humans (74). However, few studies have evaluated the role of miR-671. This analysis implicated this miRNA in neuronal apoptosis (75), in the inflammatory response in colonic epithelial cells (76) and in cancers (77, 78). Furthermore, no reports in the literature have described altered expression of miR-193b and miR-671 in the same context. In this study, we identified both miR-193b and miR-671 as relevant regulators of the inflammatory response observed in human LCL patients.

Our findings revealed six innate immune pathways common to both data sets: communication between innate and adaptive immune cells; granulocyte adhesion and diapedesis; crosstalk between dendritic cells and natural killer cells; dendritic cell maturation; TREM1 signaling; and agranulocyte adhesion and diapedesis. Most of these pathways have been described in the literature as important to the pathogenesis of human LCL (79–81), except for TREM1 signaling (Triggering Receptor Expressed on Myeloid cells). This pathway is a potent amplifier of the pro-inflammatory innate immune response, which should improve pathogen detection and killing. However, the excessive production of inflammatory mediators can also injure host tissues, leading to immunopathology (82). In this context, mice deficient in *TREM1* exhibit reduced inflammation and lesion size upon infection by *L. major* (83), suggesting a pathogenic impact of TREM-1 signaling. Similar results were observed for mice infected with influenza virus or *Legionella pneumophila* (83). Furthermore, it has been demonstrated that, after *in vitro* stimulation with *L. braziliensis*, TREM1 signaling is modulated only in cells from LCL patients who are high producers of IFN-γ (6). Together, these findings reveal the pathogenic potential of TREM-1 signaling but also indicate that its regulation by endogenous miRNAs could be promising in preventing excessive inflammation while preserving pathogen control. The potential of TREM-1 as a therapeutic target was already demonstrated for experimental colitis (84), influenza infection and LCL caused by *L. major* (83). However, the identity of endogenous agonists/antagonists of TREM-1 has not been completely resolved. The present study identifies miR-193b as a candidate to antagonize TREM-1 expression under inflammatory conditions in an endogenous manner. Ongoing experiments from our group will define the pathogenic role of TREM-1 in patients with LCL caused by *L. braziliensis* that could ultimately determine disease outcome and prognosis. In addition, manipulation of the miR-193b and TREM-1 axis could also be useful for other inflammatory diseases, since the mechanism by which TREM-1 promotes inflammation seems to be common for distinct models of immune-mediated illness.

Several molecules are involved in inflammatory processes, such as different surface receptors, cytokines, and chemokines, which can also be targeted by miRNAs (85–87). In addition to TREM-1, we observed a strong correlation between miR-193b/CD40 and miR-671/TNFR, and both receptors are considered essential in mediating a variety of immune and inflammatory responses (88–94). Signaling through CD40 and TNFR cooperate to induce anti-*Leishmania* activity and inflammation (95). However, excessive expression of these molecules can lead to tissue injury and exacerbate immunopathology (96–98). Anti-TNF therapy, which limits TNFR signaling, has been tested in mouse models for different inflammatory diseases, such as rheumatoid arthritis, psoriasis, and bowel diseases (99–101). In this way, the search for new strategies to regulate TNFR expression should include miRNAs. Promising data were published in a mouse model of inflammatory shock where miR-511 was induced to downregulate TNFR protein expression, whereas its target mRNA remained unaltered (98). Silencing the CD40 pathway has also become an attractive target to reduce inflammation in different disease models (97, 102–104). Under inflammatory conditions, endothelial cells downregulate the expression of miR-424 and miR-503, which are both related with the upregulation of CD40. However, treatment of these cells with pioglitazone, a PPARg agonist, activates the transcription of miR-424 and miR-503 and leads to suppression of CD40 expression, suggesting that miRNAs may act as key modulators of the endothelial inflammatory response (97). Together, these findings indicate that a similar approach might be applied in the context of Cutaneous Leishmaniasis, where the altered expression of miR-193b and miR-671 could be reverted in order to downregulate TNFR expression. Further experiments are necessary to clarify if this could help to balance the inflammatory response and minimize tissue injury.

Molecules with significantly altered expression could also be used as markers for diagnosis, disease progression, response to treatment or clinical outcome, contributing to clinical management and improving patient quality of life (105–108). Several studies have shown that the use of biomarkers, including miRNAs, improves diagnostic efficiency (109–112). Since miRNA expression has been shown to be stable and tissue-specific, miRNAs are potential biomarkers in the analysis of tissue and body fluids (113–115). Prediction analysis revealed that the expression profile of miR-200b and miR-200c is able to discriminate patients with prostate cancer from HCs and define the risk of disease in predisposed individuals. These results were validated in serum samples and showed higher specificity and sensibility then PSA (Prostate-specific antigen) testing (112). Similar results were also obtained for different types of cancer, where circulating miRNAs are potential markers of disease progression and response to treatment, as well as a promising screening test for cancer detection (12, 116–118). However, there is a lack of studies suggesting the potential of miRNAs as markers of infectious/parasitic diseases in humans. The majority of these studies have focused on tuberculosis, sepsis, and viral infections (such as HCV, HBV, and HIV) and compared the profiles of circulating miRNAs between patients and HCs (20, 45, 119, 120). Few studies have suggested that *S. mansoni-*derived miR-277, miR-3479, and bantam (a specific *Schistosoma* sp. miRNA) are potential markers for parasitic infection, in mouse and human serum, in order to evaluate the response to treatment (48). Furthermore, in the mouse model of *T. gondii* infection, miR-712, miR-511, and miR-217 have been indicated as a potential miRNA signature that can be useful for early detection of infection (49). In addition, a profile of deregulated miRNAs in malaria patients with multiple organ failure indicated that miRNAs can be used as biomarkers of disease severity (52). Together, these studies reveal that miRNAs can also be useful tools in the management of infectious diseases.

Regarding *Leishmania* infection in humans, our study provides evidence that the healing time of patients with LCL can be associated with the correlation between miRNAs and theirs target genes. We observed that the axis of miR-193b, miR-671, and TREM-1 could be used as a predictive marker of prognosis for human LCL. Further studies are necessary to define whether this miRNA expression profile can be applied as a biosignature for better prognosis, allowing an early definition of the most effective therapy of human LCL. In summary, we revealed in this study a previously unknown expression profile of miRNAs involved in the regulation of pathways that are important for the immunopathogenesis of human LCL, shedding new light into the mechanisms of disease severity and prognosis. It is tempting to speculate that the axis of miR-193b, miR-671, and TREM-1 is a potential candidate to the development of new tools to manage this disease and other inflammatory skin disorders.

## Ethics Statement

This study was carried out in accordance with the recommendations of Institutional Review Board of the Gonçalo Moniz Institute, Bahia-Brazil with written informed consent from all subjects. All subjects gave written informed consent in accordance with the Declaration of Helsinki. The protocol was approved by the Institutional Review Board of the Gonçalo Moniz Institute, Bahia-Brazil (license number: 47120215.8.0000.0040).

## Author Contributions

SN contributed to the design and conception of the experiments, conducted molecular biology experiments, analyzed and interpreted data, and drafted the manuscript. SN, IS, and MA performed RT-qPCR assays and helped with quality control and analysis. SN, PR, CB, and PO normalized the data and performed all statistical analyses. AN, LS, TC, RK, VB, and AB provided biopsies skin samples. SN, PO and NT conceived the study and participated in its design, helped to analyze and interpret the data, and drafted the manuscript.

## Conflict of Interest Statement

The authors declare that they have no commercial or financial relationships that could be construed as a potential conflict of interest.

## Footnotes

^1^www.ncbi.nlm.nih.gov/geo/.

^2^www.ensembl.org/biomart/.

^3^www.mev.tm4.org.

^4^www.qiagenbioinformatics.com/products/ingenuity-pathway-analysis/.

^5^www.targetscan.org.

## Supplementary Material

Table S1 Demographic and clinical metadata from *Leishmania braziliensis* patients.

Table S2 MicroRNA probes identified on the Illumina HT 12 v4 platform (GSE55664 data set).

Table S3 MicroRNA probes identified on the Agilent SurePrint GE Human G3v2 platform (GSE63931 data set).

Table S4 Identification of known microRNAs after data refinement.

Table S5 Raw datasets of identified canonical pathways related to LCL found on Ingenuity Pathways Analysis.

Table S6 Canonical pathways of the innate immune response that are significantly modulated in leishmaniasis as identified by Ingenuity Pathways Analysis.

Figure S1 Pipeline of the search for public transcriptome data sets of human cutaneous leishmaniasis. Microarray data sets for experiments performed on human skin biopsies of cutaneous leishmaniasis caused by *Leishmania braziliensis* were searched at the Gene Expression Omnibus database (www.ncbi.nlm.nih.gov/geo/). Two data sets were found [GSE55664 (Illumina HT 12 v4 platform) and GSE63931 (Agilent SurePrint GE Human G3v2 platform)]. The Ensembl/BioMart tool was applied for data mining, and the numbers of microRNA probes found were 792 and 141 for GSE55664 and GSE63931, respectively.

Figure S2 Unsupervised clustering of healthy and cutaneous leishmaniasis skin biopsies based on microRNA expressions. Hierarchical clustering was performed using the Euclidean distance method for sets of miRNA probes expressed in healthy control (HC; orange bar) and in localized cutaneous leishmaniasis (LCL; brown bar) skin samples. **(A)** Heat map showing the expression profiles of 792 microRNA probes from the GSE55664 (Illumina HT12 v4 platform) data set in 10 HC and 25 LCL samples. **(B)** Heat map showing the expression profiles of 141 microRNA probes from the GSE63931 (Agilent Sure Print GE Human G3v2 platform) data set in eight HC and eight LCL samples. Rows represent microRNA probes and columns represent samples. The scale bar symbolizes the intensity of expression for each microRNA probe (Log2).

Figure S3 Pipeline for the identification of pathways related to immune response in LCL. Both LCL datasets, GSE55664 and GSE63931, were analyzed by Ingenuity Pathways Analysis to identify the canonical pathways related to innate immune response during this disease. Molecules composing these pathways with differential expression (−2< FC >2 and *p*-value < 0.05) were selected. Using the Targetscan tool, these molecules were searched as targets for the microRNAs found in LCL.

Figure S4 High confidence microRNAs deposited in miRBase 21 database and available in each platform. **(A)** Total number of *Homo sapiens* microRNA precursors considered high or low confidence, as described in miRBase 21 (www.mirbase.org/). **(B)** Absolute number (#) of high (black bars) or low (gray bars) confidence microRNA probes. Some probes covered putative microRNAs not deposited in miRBase 21 (white bars).

Figure S5 Number of correlations between microRNAs and their target genes according to different response to treatment. Total number of correlations between miRNAs and their target genes within groups defined based on the response to treatment. This analysis quantifies the number of correlations and compares among healthy control individuals (CTR), LCL patients cured up to 59 days (0–59), 60–89 days (60–89), or more than 90 days (>90).
