# Supplementary figures and images for: Integrated Analysis Reveals That miR-193b, miR-671, and TREM-1 Correlate With a Good Response to Treatment of Human Localized Cutaneous Leishmaniasis Caused by Leishmania braziliensis
Source: Front Immunol. 2018 Apr 4;9:640. doi: 10.3389/fimmu.2018.00640 (PMC5893808; doi:10.3389/fimmu.2018.00640)

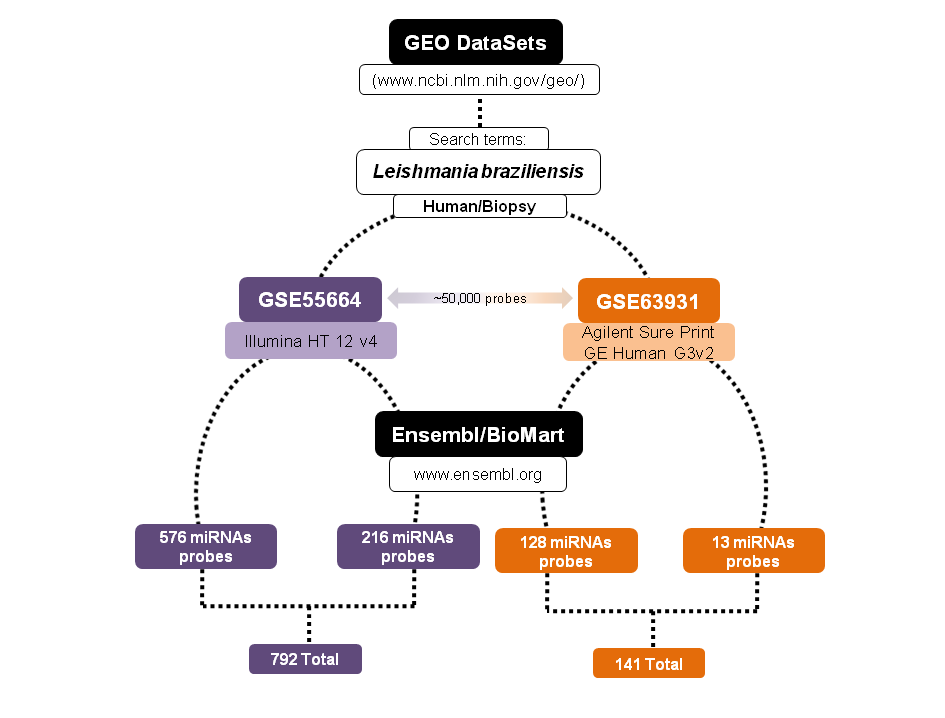

Supplement: Figure S1 — Pipeline of the search for public transcriptome data sets of human cutaneous leishmaniasis. Microarray data sets for experiments performed on human skin biopsies of cutaneous leishmaniasis caused by Leishmania braziliensis were searched at the Gene Expression Omnibus database (www.ncbi.nlm.nih.gov/geo/). Two data sets were found [GSE55664 (Illumina HT 12 v4 platform) and GSE63931 (Agilent SurePrint GE Human G3v2 platform)]. The Ensembl/BioMart tool was applied for data mining, and the numbers of microRNA probes found were 792 and 141 for GSE55664 and GSE63931, respectively. [file Image_1.tif]

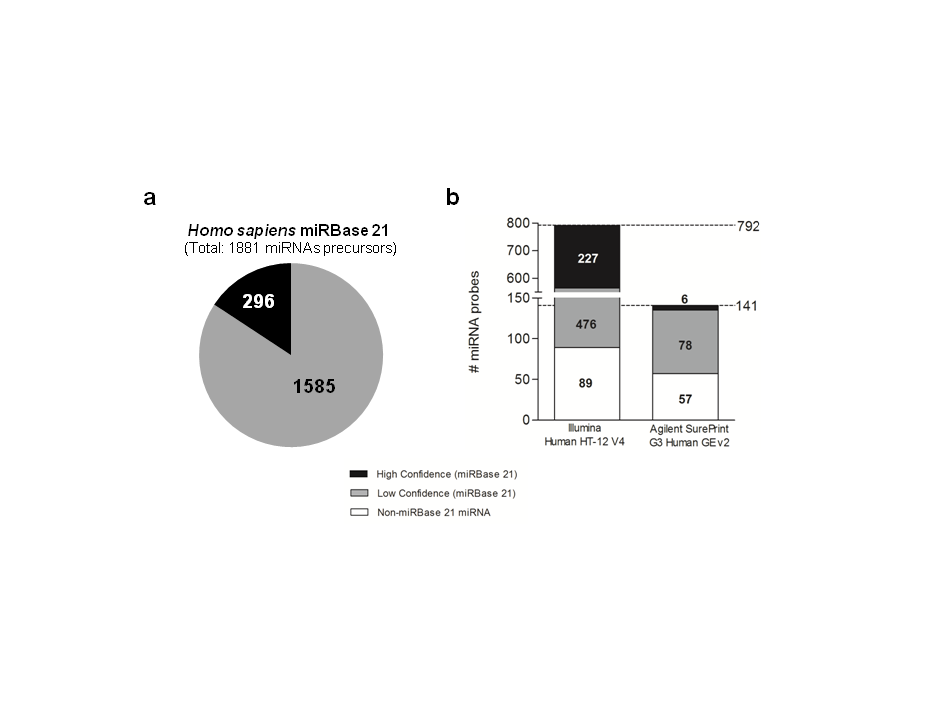

Supplement: Figure S2 — Unsupervised clustering of healthy and cutaneous leishmaniasis skin biopsies based on microRNA expressions. Hierarchical clustering was performed using the Euclidean distance method for sets of miRNA probes expressed in healthy control (HC; orange bar) and in localized cutaneous leishmaniasis (LCL; brown bar) skin samples. (A) Heat map showing the expression profiles of 792 microRNA probes from the GSE55664 (Illumina HT12 v4 platform) data set in 10 HC and 25 LCL samples. (B) Heat map showing the expression profiles of 141 microRNA probes from the GSE63931 (Agilent Sure Print GE Human G3v2 platform) data set in eight HC and eight LCL samples. Rows represent microRNA probes and columns represent samples. The scale bar symbolizes the intensity of expression for each microRNA probe (Log2). [file Image_2.tif]

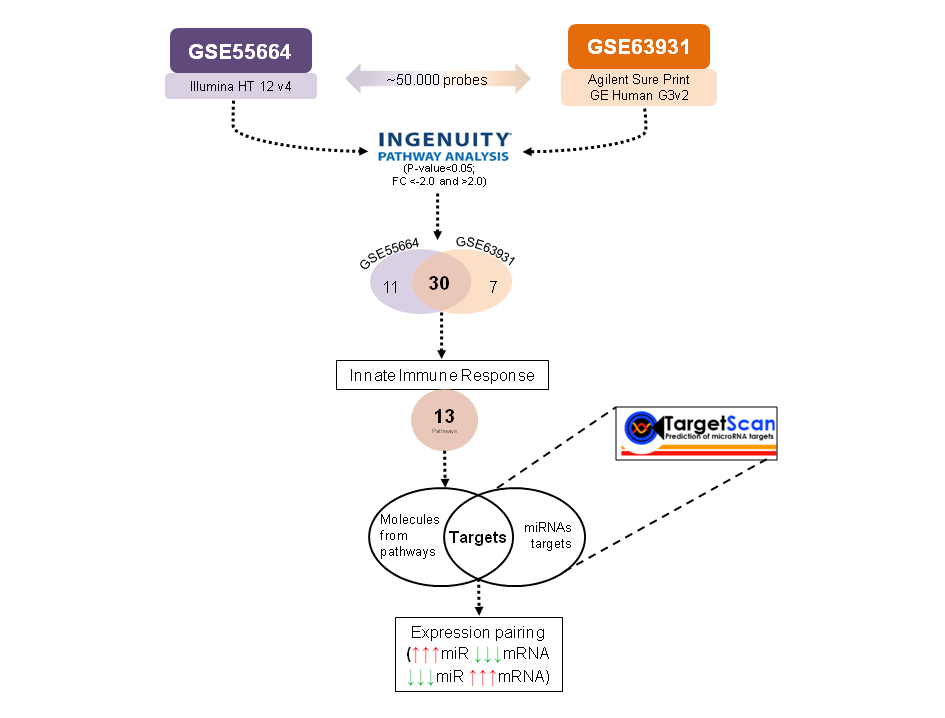

Supplement: Figure S3 — Pipeline for the identification of pathways related to immune response in LCL. Both LCL datasets, GSE55664 and GSE63931, were analyzed by Ingenuity Pathways Analysis to identify the canonical pathways related to innate immune response during this disease. Molecules composing these pathways with differential expression (−2< FC >2 and p-value < 0.05) were selected. Using the Targetscan tool, these molecules were searched as targets for the microRNAs found in LCL. [file Image_3.tif]

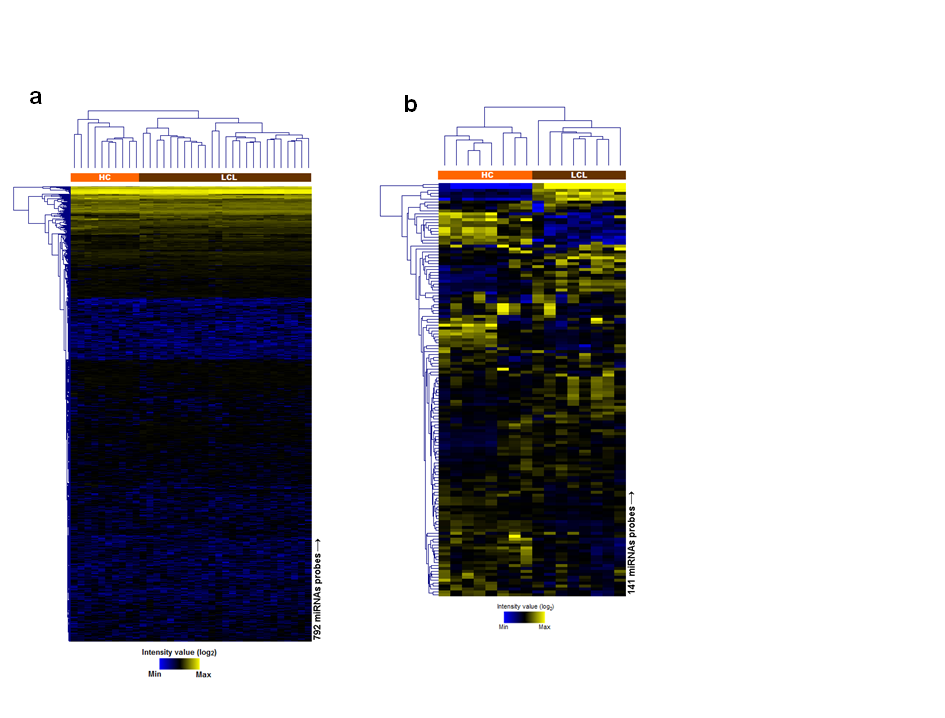

Supplement: Figure S4 — High confidence microRNAs deposited in miRBase 21 database and available in each platform. (A) Total number of Homo sapiens microRNA precursors considered high or low confidence, as described in miRBase 21 (www.mirbase.org/). (B) Absolute number (#) of high (black bars) or low (gray bars) confidence microRNA probes. Some probes covered putative microRNAs not deposited in miRBase 21 (white bars). [file Image_4.tif]

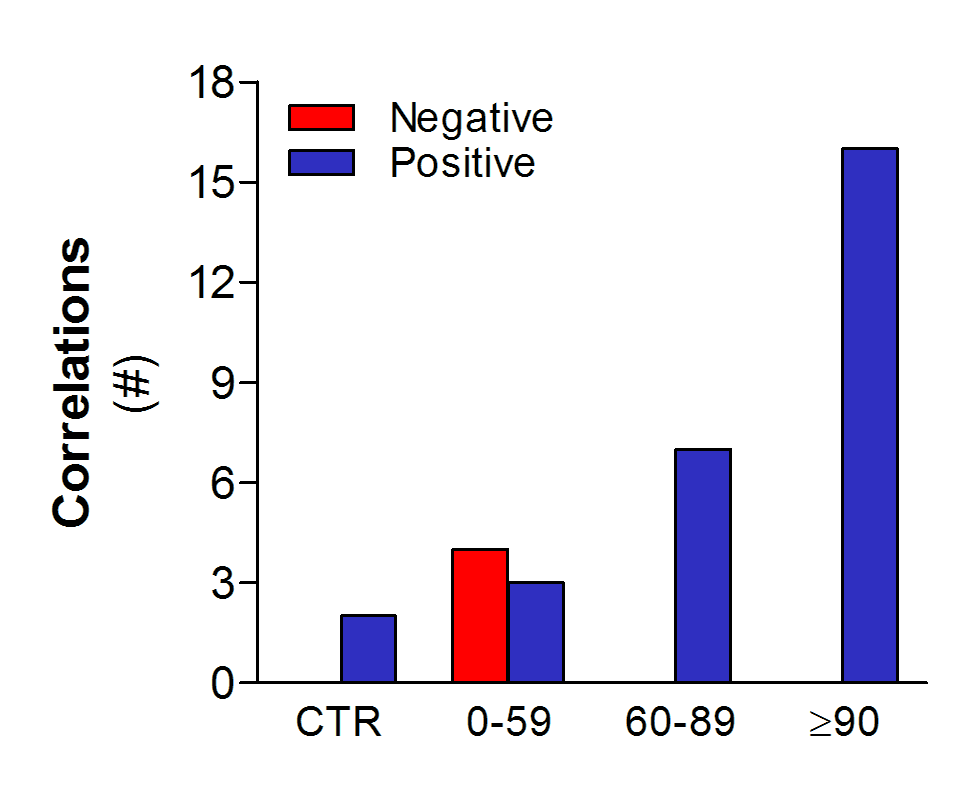

Supplement: Figure S5 — Number of correlations between microRNAs and their target genes according to different response to treatment. Total number of correlations between miRNAs and their target genes within groups defined based on the response to treatment. This analysis quantifies the number of correlations and compares among healthy control individuals (CTR), LCL patients cured up to 59 days (0–59), 60–89 days (60–89), or more than 90 days (>90). [file Image_5.tif]
